# Effect of surface carbonates on the cyclability of LiNbO_3_-coated NCM622 in all-solid-state batteries with lithium thiophosphate electrolytes

**DOI:** 10.1038/s41598-021-84799-1

**Published:** 2021-03-08

**Authors:** A-Young Kim, Florian Strauss, Timo Bartsch, Jun Hao Teo, Jürgen Janek, Torsten Brezesinski

**Affiliations:** 1 Battery and Electrochemistry Laboratory, Institute of Nanotechnology, Karlsruhe Institute of Technology (KIT), Hermann-von-Helmholtz-Platz 1, 76344 Eggenstein-Leopoldshafen, Germany; 2 Institute of Physical Chemistry & Center for Materials Science, Justus-Liebig-University Giessen, Heinrich-Buff-Ring 17, 35392 Giessen, Germany; 3 Present Address: VARTA AG, Alfred-Krupp-Str. 9, 73479 Ellwangen, Germany; 4 Present Address: Mercedes-Benz Korea Ltd., Seoul, Republic of Korea

**Keywords:** Batteries, Batteries, Synthesis and processing

## Abstract

While still premature as an energy storage technology, bulk solid-state batteries are attracting much attention in the academic and industrial communities lately. In particular, layered lithium metal oxides and lithium thiophosphates hold promise as cathode materials and superionic solid electrolytes, respectively. However, interfacial side reactions between the individual components during battery operation usually result in accelerated performance degradation. Hence, effective surface coatings are required to mitigate or ideally prevent detrimental reactions from occurring and having an impact on the cyclability. In the present work, we examine how surface carbonates incorporated into the sol–gel-derived LiNbO_3_ protective coating on NCM622 [Li_1+*x*_(Ni_0.6_Co_0.2_Mn_0.2_)_1–*x*_O_2_] cathode material affect the efficiency and rate capability of pellet-stack solid-state battery cells with β-Li_3_PS_4_ or argyrodite Li_6_PS_5_Cl solid electrolyte and a Li_4_Ti_5_O_12_ anode. Our research data indicate that a hybrid coating may in fact be beneficial to the kinetics and the cycling performance strongly depends on the solid electrolyte used.

## Introduction

Li-ion batteries (LIBs) using liquid organic electrolytes play a pivotal role in our modern society, especially for powering portable electronic devices and electric vehicles^1–3^. The demand for energy storage is growing strongly, largely driven by the automotive industry. Substituting an inorganic (superionic) solid electrolyte for the liquid electrolyte in LIBs is a potentially viable strategy to increase energy density and minimize safety risks due to cell failure^4–6^. Lithium thiophosphates, such as argyrodite Li_6_PS_5_Cl, are among the most promising solid electrolytes because of their high ionic conductivity at room temperature and favorable ductility properties^7–17^. At the positive electrode side, Ni-rich layered lithium metal oxides, such as LiNi_1–*x*–*y*_Co_*x*_Mn_*y*_O_2_ (NCM or NMC) or LiNi_1–*x*–*z*_Co_*x*_Al_*z*_O_2_ (NCA) with ≥ 0.6 Ni content, are regarded generally as state-of-the-art cathode materials for bulk solid-state battery (SSB) applications^18–21^, as in the case of energy-dense LIBs. However, combining such cathode materials with lithium thiophosphate solid electrolytes is hampered by side reactions at the interfaces during electrochemical cycling, leading to low reversibility and impedance buildup and therefore to performance decay^22–27^. Hence, in order to achieve stable cycling of the cathode, the outer surface of the storage particles needs to be covered by a protective layer^28–30^, with Li-based oxides being the most widely studied coating materials (e.g., LiNbO_3_^31^, LiTaO_3_^32^, Li_2_ZrO_3_^33^, Li_4_Ti_5_O_12_^34^ or Li_2_CO_3_^35^). One that stands out and has been proven to be an effective coating material is LiNbO_3_ (apparently because of favorable charge-transport properties in the amorphous state)^36^. Although reasonably stable cycling has been achieved in the past with various coating chemistries, in-depth understanding of the functionality of coatings for active/inactive electrode materials is still lacking. Ultimately, scalable and cost-efficient methods that are capable of depositing ultrathin and conformal coatings on arbitrary substrates are urgently required (note that atomic layer deposition is one such technique with some limitations)^28^.

Recently, it has been shown that artificially formed Li_2_CO_3_ positively affects the cycling stability of SSB cells, especially when combined with Li_3_BO_3_^37^ or LiNbO_3_^38^ in a kind of hybrid or solid-solution coating on layered lithium metal oxide cathode materials. However, the influence that the amount of carbonate incorporated into the protective surface layer has on the cycling performance is still unclear at present. Here, we study the effect of carbonate content by varying the Li:Nb molar ratio during application of the coating onto NCM622 cathode material (in an attempt to tailor the coating composition). The surface-treated samples were characterized using different methods and electrochemically tested in pellet-stack SSB cells with a Li_4_Ti_5_O_12_ (LTO) anode and either β-Li_3_PS_4_ or Li_6_PS_5_Cl as solid electrolyte.

## Results

The surface coatings on NCM622 were prepared by the sol–gel method using ethoxide precursors, followed by heating at 300 °C in air. The molar ratio of Li:Nb was varied from 0.5 to 3.0 in the synthesis and the resultant samples are denoted hereafter as S1 (0.5), S2 (0.75), S3 (1.0), S4 (2.0) and S5 (3.0), with the number in the brackets referring to the actual molar ratio. Note that 1.0, in principle, corresponds to the composition of stoichiometric LiNbO_3_. In addition, the sample prepared using a Li:Nb molar ratio of 1.0 was heated in oxygen flow to produce a LiNbO_3_-coated NCM622 that is lean in carbonate species (referred to as S6).

**Figure 1:**
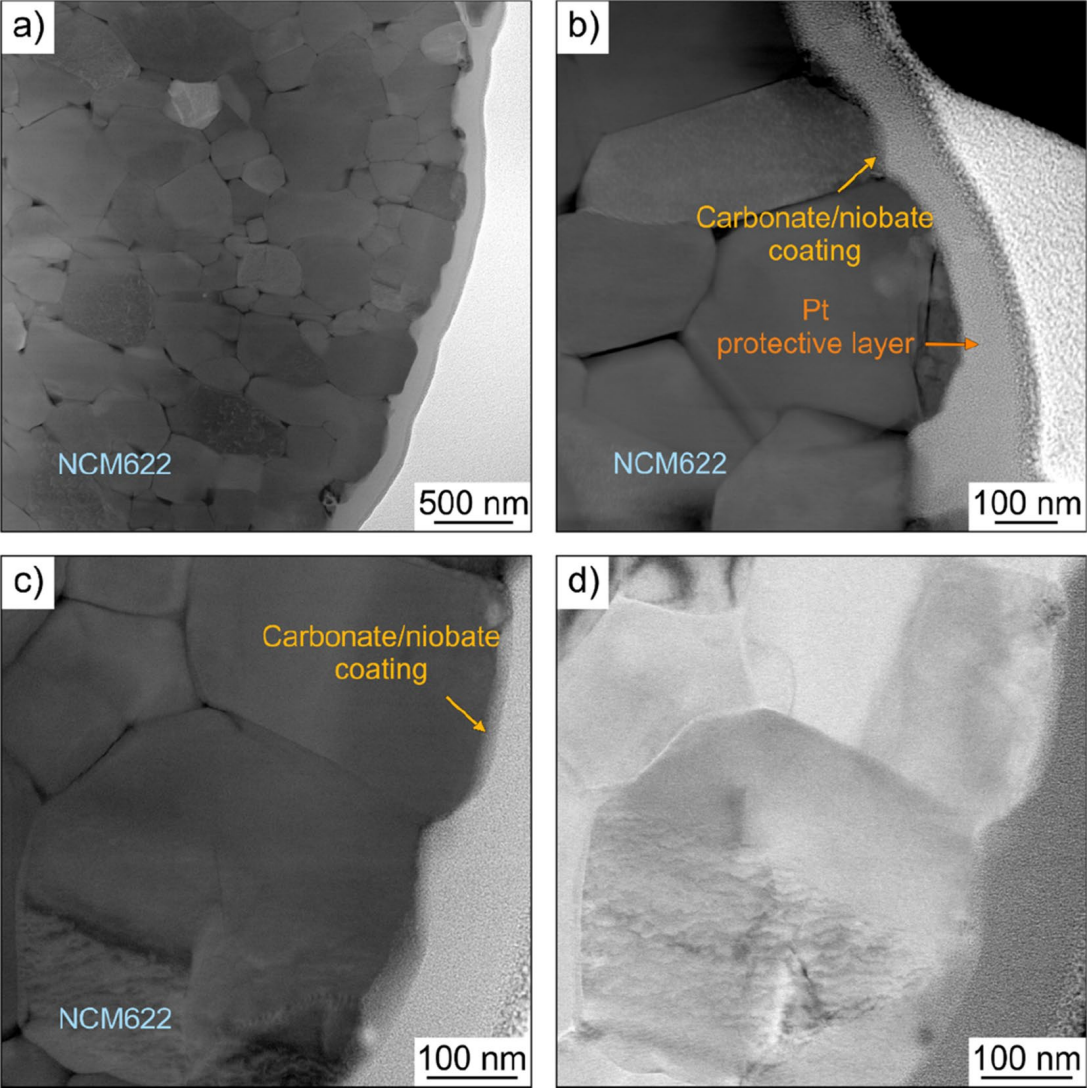
(**a**–**c**) High-angle annular dark-field and (**d**) bright-field STEM images of the coated NCM622 cathode material (sample S3).

Figure 1a–d shows scanning transmission electron microscopy (STEM) images at different magnifications of the coated NCM622 cathode material (sample S3). As can be seen, the secondary particles (*d*_90_ = 6.0 μm) consisted of primary particles of size less than 400 nm and the top surface was covered by a continuous layer, up to a few tens of nanometers thick. The fact that the oxide coating contains niobium and carbon has been demonstrated previously by energy-dispersive X-ray spectroscopy (EDS) mapping and electron energy loss spectroscopy (EELS) under cryo conditions^38^. The results of the latter study also suggest the formation of a hybrid coating rather than individual domains of sol–gel-derived LiNbO_3_ and Li_2_CO_3_ due to reaction with CO_2_ from the air at elevated temperatures. The microscopy data in Fig. 1 corroborate these findings (see also high-magnification STEM image and EDS line scan in Fig. S1 in the Supporting Information).

**Figure 2:**
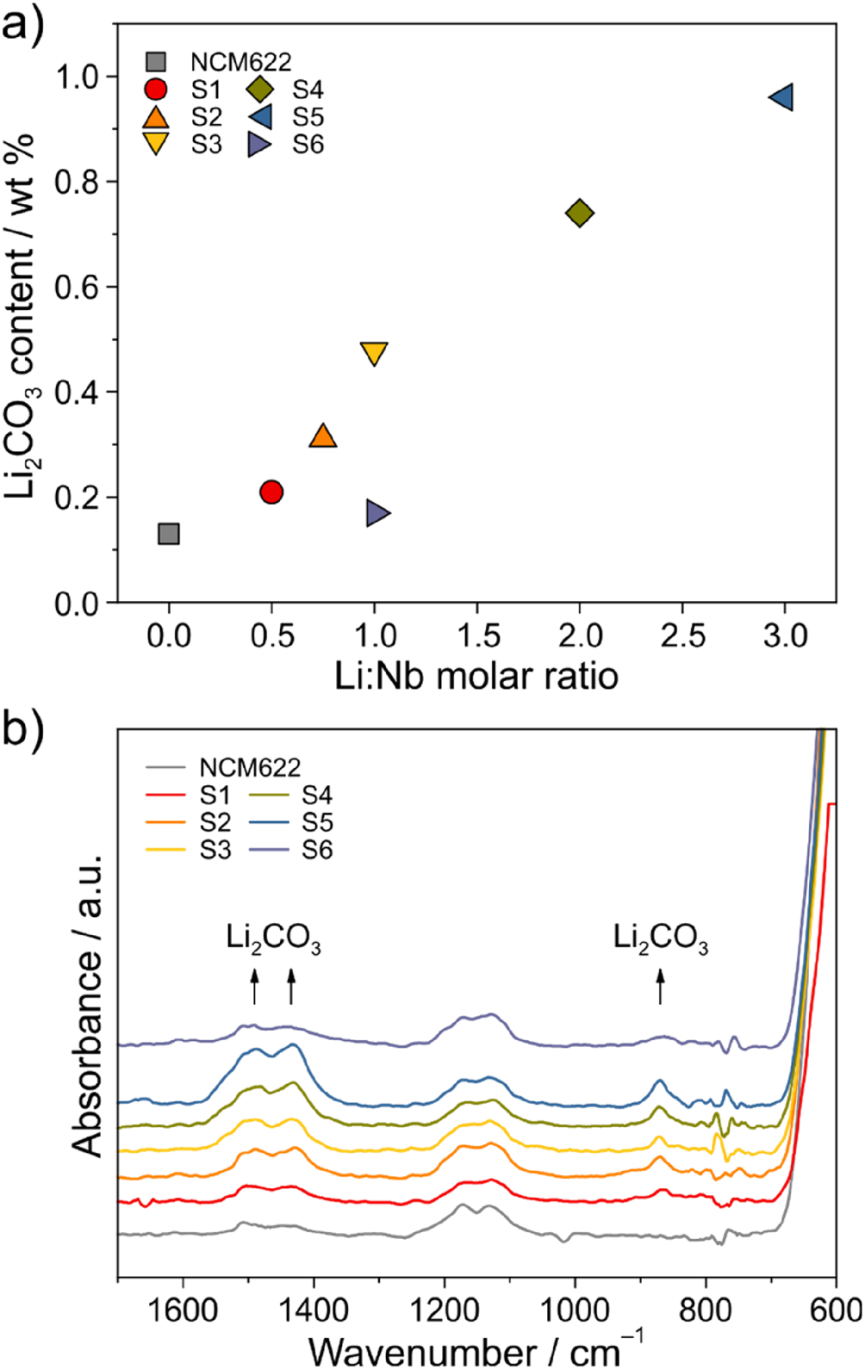
(**a**) Li_2_CO_3_ content of the uncoated (pristine) and coated NCM622 cathode materials from acid titration measurements and (**b**) the corresponding background-corrected ATR-IR spectra.

The carbonate content was quantified by acid titration measurements coupled with mass spectrometry (Fig. 2a). In general, the higher the molar ratio of Li:Nb, the more carbonate (referred to as Li_2_CO_3_ herein) formed upon heating in air. For S1 to S5, the Li_2_CO_3_ content increased virtually linearly from ~ 0.2 to ~ 0.95 wt%, indicating that the residual Li was consumed in the formation of surface carbonates. For comparison, uncoated (pristine) NCM622 cathode material exhibited ~ 0.1 wt%. Moreover, for the material heated in oxygen (S6), a much lower Li_2_CO_3_ content was found as compared to S3 (~ 0.15 versus ~ 0.5 wt%), which underwent post treatment in air.

Using attenuated total reflection-infrared (ATR-IR) spectroscopy, bands at ~ 1490/1430 cm^–1^ and ~ 870 cm^–1^ due to carbonate stretching and deformation vibrations, respectively, were detected (Fig. 2b)^39^. The bands at ~ 1170 and ~ 1130 cm^–1^ can be assigned to the NCM622 cathode material itself. As expected, they were virtually identical for all of the samples studied here. The increase in intensity of the carbonate vibrational bands seen for S1 to S5 is consistent with the trend in Li_2_CO_3_ content from the acid titration measurements.

The amount of Nb in the uncoated and coated NCM622 cathode materials was determined by inductively coupled plasma-optical emission spectroscopy (ICP-OES). The Nb content (~ 0.6 wt%, equivalent to ~ 1.0 wt% LiNbO_3_ [specifically for S3 to S6]) was similar for the coated samples. Hence, hypothetically, the formation of LiNbO_3_ and Li_2_CO_3_ would lead to total weight fractions of less than 1.3 wt% for S1, S2 and S6 and ~ 1.5 to ~ 2.0 wt% for S3 to S5 (Table S1).

Overall, the results from ICP-OES and acid titration measurements imply that the thickness of the coating increases with increasing Li:Nb molar ratio. However, it is not expected to vary significantly among the samples. The same holds for the quality of the protective layer. Nevertheless, we note that the uniformity of surface coatings strongly depends on the method of deposition and the sol–gel process used in this work has been shown to provide only limited control over conformality and coating thickness^28,38,40^. Hence, it is reasonable to assume that the hybrid coating examined here is not uniform in thickness throughout (especially when also considering the STEM imaging data in Figs. 1 and S1).

**Figure 3:**
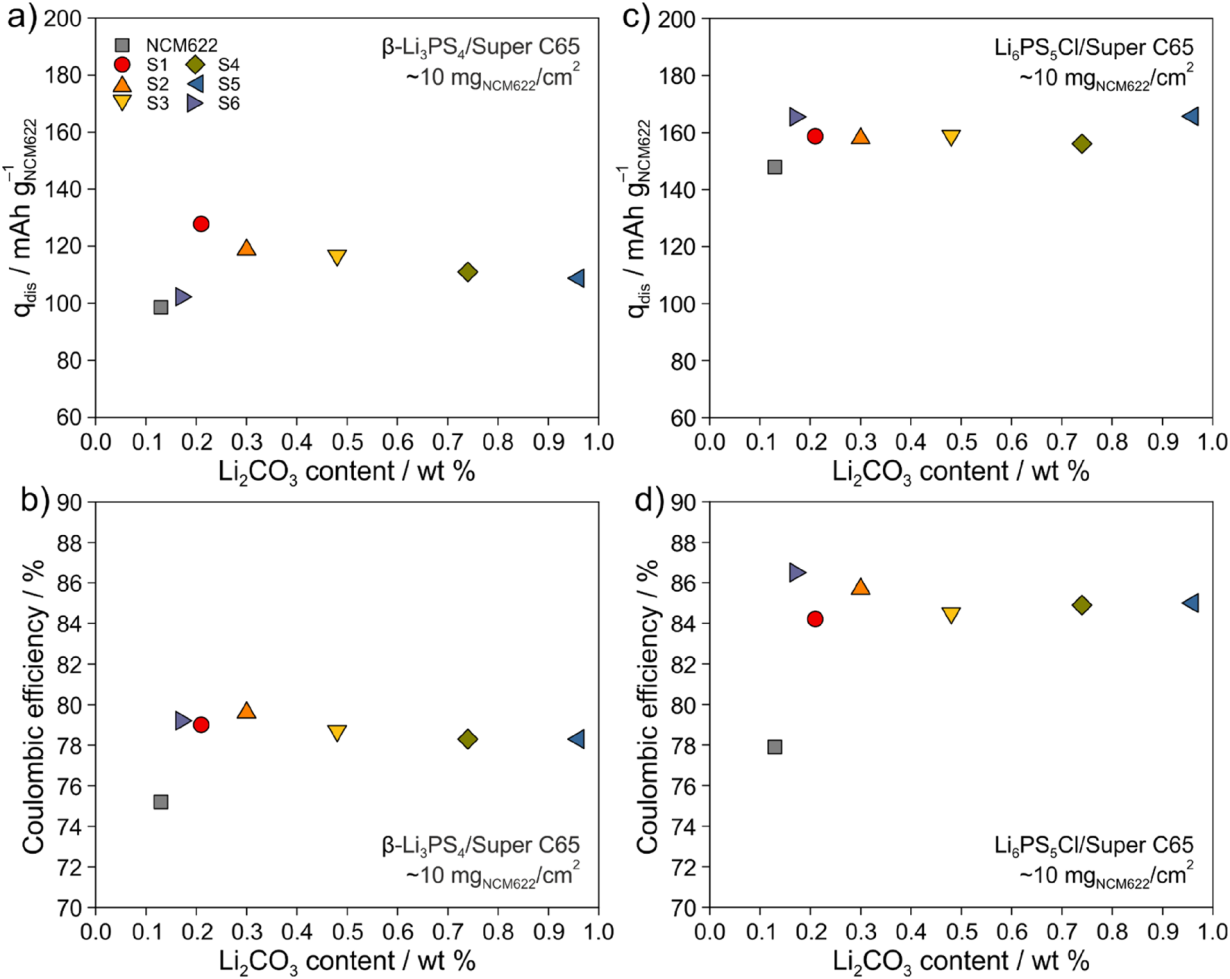
Initial specific discharge capacity and Coulombic efficiency at C/10 rate and 25 °C of SSB cells using the uncoated (pristine) or coated NCM622 cathode materials and (**a**, **b**) β-Li_3_PS_4_/Super C65 carbon black or (**c**, **d**) Li_6_PS_5_Cl/Super C65 carbon black. The anode was made of LTO, β-Li_3_PS_4_ or Li_6_PS_5_Cl solid electrolyte and Super C65 carbon black additive.

As mentioned above, the different NCM622 cathode materials were electrochemically tested in pellet-stack SSB cells with an LTO anode in the voltage range between 1.35 and 2.85 V vs Li_4_Ti_5_O_12_/Li_7_Ti_5_O_12_. To this end, cathode composites were prepared by milling mixtures 7:2.9:0.1 by weight of NCM622, β-Li_3_PS_4_ or Li_6_PS_5_Cl and Super C65 carbon black. The areal loading was ~ 10 mg_NCM622_/cm^2^. Note that carbon black was used as a conductive additive to promote kinetics (counteract sluggish charge transfer) by improving electronic transport^41^. Figure 3a shows the initial specific discharge capacity at a rate of C/10 (1C = 180 mA/g_NCM622_) and 25 °C of cells using the β-Li_3_PS_4_-based cathode composites. Uncoated NCM622 delivered a capacity of ~ 99 mAh/g_NCM622_. Applying a carbonate-deficient LiNbO_3_ coating to the NCM622 cathode material (sample S6) resulted in a minor increase to ~ 102 mAh/g_NCM622_. However, as can be seen from Fig. 3b, the initial Coulombic efficiency increased significantly from ~ 75% for uncoated NCM622 to ~ 79% for S6. S1 achieved the largest first-cycle specific discharge capacity (~ 128 mAh/g_NCM622_) of any sample tested. For S2 to S5, the capacity decreased rather linearly with increasing carbonate content, from ~ 119 to ~ 109 mAh/g_NCM622_. Considering that the initial Coulombic efficiencies were similar at ~ 79%, this result may be related to the fact that Li_2_CO_3_ is electronically insulating. Because electronic limitations were somewhat mitigated by the use of Super C65 carbon black, it seems that the relatively low initial specific discharge capacities are due to both the moderate room-temperature ionic conductivity (~ 0.2 versus ~ 1.8 mS/cm for Li_6_PS_5_Cl) and poor electrochemical stability of β-Li_3_PS_4_. However, it should be noted that the theoretical anodic stability of β-Li_3_PS_4_ and Li_6_PS_5_Cl is about the same, but apparently the degree of passivation by the decomposition interphases is different^42–44^.

In order to prove/disprove this hypothesis, Li_6_PS_5_Cl was substituted for β-Li_3_PS_4_ in the SSB cells. In fact, the Li_6_PS_5_Cl-based cathode composites achieved both much higher first-cycle specific discharge capacities (Fig. 3c) and Coulombic efficiencies (Fig. 3d) at C/10 rate and 25 °C. For uncoated NCM622, in particular, the capacity increased by more than 40 mAh/g_NCM622_. The cells with the coated samples delivered initial specific discharge capacities of (161 ± 5) mAh/g_NCM622_, equivalent to (1.61 ± 0.05) mAh/cm^2^, and showed similar Coulombic efficiencies of ~ 85%, compared to ~ 78% for uncoated NCM622. These results emphasize the benefits of higher ionic conductivity and improved interfacial stability of the argyrodite solid electrolyte.

This is also evident from the voltage profiles and the corresponding differential capacity plots (Fig. S2). Unlike for the Li_6_PS_5_Cl cells, the first-cycle charge/discharge curves of the β-Li_3_PS_4_ cells differed significantly from one another, thereby indicating that the β-Li_3_PS_4_-based cathode composites are more susceptible to side reactions (electrochemical degradation). The latter cells also showed a significant voltage drop at the beginning of the discharge cycle. In case of the Li_6_PS_5_Cl cells, the difference in discharge capacity only occurred below ~ 2.0 V vs Li_4_Ti_5_O_12_/Li_7_Ti_5_O_12_. Overall, these results suggest that the charge transfer across the β-Li_3_PS_4_|NCM622 interface is hindered during charging and discharging. The large differences in overpotential between the β-Li_3_PS_4_ and Li_6_PS_5_Cl cells (~ 250 versus ~ 50 mV voltage hysteresis) can be seen even more clearly from the differential capacity plots (Fig. S2). For example, for those using sample S3, the mean charge/discharge voltages of the initial cycle were ~ 2.40/2.12 and ~ 2.32/2.27 V vs Li_4_Ti_5_O_12_/Li_7_Ti_5_O_12_, respectively. We hypothesize that this is also associated with the different chemical nature of the as-formed interphases.

**Figure 4:**
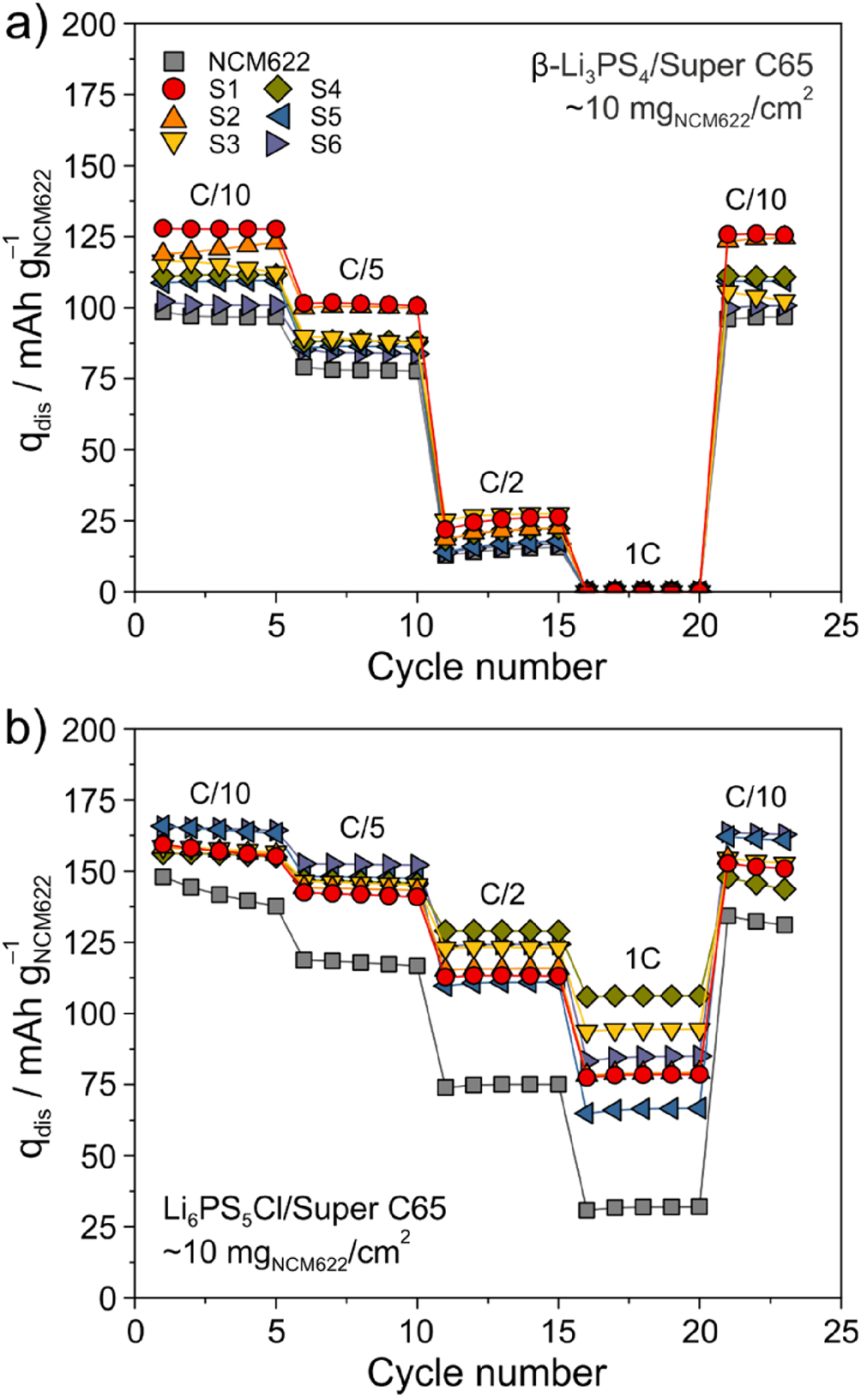
Rate performance at 25 °C of SSB cells using the uncoated (pristine) or coated NCM622 cathode materials and (**a**) β-Li_3_PS_4_/Super C65 carbon black or (**b**) Li_6_PS_5_Cl/Super C65 carbon black. The anode was made of LTO, β-Li_3_PS_4_ or Li_6_PS_5_Cl solid electrolyte and Super C65 carbon black additive.

Finally, the rate capability was studied at 25 °C to gain some insights into kinetic limitations. Specifically, the C-rate was increased every five cycles from C/10 to C/5, C/2 and 1C and then it was decreased back to C/10 for three more cycles. Figure 4a shows the respective data for SSB cells using the β-Li_3_PS_4_-based cathode composites. As can be seen, they exhibited stable cycling behavior, with mostly minor capacity degradation after 23 cycles. The same trend of decreasing specific discharge capacity with increasing carbonate content for the initial cycle was evident at C/5. S1 and S2 were still capable of delivering ~ 100 mAh/g_NCM622_, compared to (83 ± 5) mAh/g_NCM622_ for all other samples. However, the capacity dropped significantly when the rate was increased to ≥ C/2 (with close-to-zero capacity at 1C). Such poor rate performance can be attributed to sluggish kinetics because of the relatively low ionic conductivity of the β-Li_3_PS_4_ solid electrolyte, among others (see ref.^45^ for a quantitative modeling study, showing that ionic conductivities of ~ 10 mS/cm are required to achieve large cell capacities at high C-rates).

Different results were obtained after Li_6_PS_5_Cl was substituted for β-Li_3_PS_4_ (Fig. 4b). Except for uncoated NCM622 cathode material in the first five cycles, all of the cells showed stable cyclability at low and high C-rates. In contrast to the β-Li_3_PS_4_-based cathode composites, S5 and S6 achieved the largest specific discharge capacities at C/10 and C/5 (~ 150 mAh/g_NCM622_), followed by S4 and S3. Apart from uncoated NCM622, S1 and S2 delivered the lowest capacities at C/5, which were still larger by ~ 40 mAh/g_NCM622_ compared to the cells using β-Li_3_PS_4_ solid electrolyte. Apparently, the amount of carbonate incorporated into the coating layer on NCM622 cathode material plays a secondary role at low C-rates when using a highly conductive solid electrolyte. Moreover, from the data in Fig. 4b, it can be inferred that there is a sweet spot in terms of carbonate content (~ 0.5–0.7 wt%), where the cells are capable of delivering specific discharge capacities of ≥ 100 mAh/g_NCM622_ at 1C. It should be noted, nevertheless, that the rate capability is probably affected by other parameters too, such as the coating microstructure and the interfacial chemistry (decomposition products), which may vary to some degree with composition of the protective surface layer^37,38,46,47^. However, because S6 also showed good rate performance with capacities of ~ 124 and ~ 85 mAh/g_NCM622_ at C/2 and 1C, respectively, and it is difficult to precisely control the carbonate content, heating in oxygen flow seems beneficial to keep (interfacial) variations in sample composition and uniformity at a minimum.

## Discussion

In summary, in this work we studied the effect that the amount of carbonate incorporated into the LiNbO_3_-based surface coating on NCM622 cathode material has on the cycling performance of pelletized SSB cells with an LTO anode. The carbonate content was successfully varied from ~ 0.1 to ~ 0.95 wt% by varying the Li:Nb molar ratio during synthesis and the post treatment conditions while keeping the Nb content constant. The uncoated (pristine) and coated NCM622 cathode materials were electrochemically tested at 25 °C in cells with either β-Li_3_PS_4_ or Li_6_PS_5_Cl and Super C65 carbon black as solid electrolyte and electronically conductive additive, respectively. The experimental data show clearly that the cyclability depends on the type of solid electrolyte used. This is in part because Li_6_PS_5_Cl has about an order of magnitude higher room-temperature ionic conductivity than β-Li_3_PS_4_. In general, β-Li_3_PS_4_ cells delivered much lower capacities than those using the argyrodite solid electrolyte and the cycling performance was found to be adversely affected with increasing carbonate content (decreasing capacity). In contrast, for Li_6_PS_5_Cl cells, the carbonate content played a secondary role at low C-rates. However, the presence of a certain amount of surface carbonate species proved to be beneficial to the rate capability. Nevertheless, carbonate-deficient, LiNbO_3_-coated NCM622 cathode material prepared by heating in oxygen flow showed good cycling performance too, suggesting that other effects are also at play. Note that, in general, both the nature and stability of interfaces and interphases between the electrode components and the solid electrolyte properties strongly affect the impedance response^48^. Taken together, the results confirm that carbonate-containing complex oxides do indeed have potential as advanced surface coatings on layered lithium metal oxide cathode materials for SSB applications^37,38,40^.

## Materials and methods

### Solid electrolytes

β-Li_3_PS_4_ (*Pnma* space group) with a room-temperature ionic conductivity of ~ 0.2 mS/cm was received from BASF SE. In brief, it was prepared from Li_2_S (99.9%, Sigma-Aldrich) and P_2_S_5_ (99%, Sigma-Aldrich) as precursors through a solvent-mediated route using tetrahydrofuran (THF), as described in the literature^19,49^. Argyrodite Li_6_PS_5_Cl (*F* − 43*m* space group) with a room-temperature ionic conductivity of ~ 1.8 mS/cm was prepared as follows: Stoichiometric amounts of Li_2_S, P_2_S_5_ and LiCl (> 99%, Alfa Aesar; dried at 300 °C in a vacuum prior to use) were loaded into a ball milling jar containing zirconia balls of diameter 10 mm. The ball-to-powder ratio was ~ 27:1. The mixture was first milled for 1 h at 250 rpm and then for 20 h at 450 rpm. Finally, the powder was recovered and heated for 5 h at 300 °C in a vacuum. Results from Rietveld analysis and microscopy imaging are presented in Fig. S3. Refined lattice parameters are given in Tables S2 and S3 and are in good agreement with literature data^7,49,50^.

### Surface coating

Size-tailored NCM622 cathode material was received from BASF SE, dried in a vacuum for 12 h and stored in an argon glovebox (MBraun, [O_2_] and [H_2_O] < 0.1 ppm)^19^. 1 M lithium ethoxide solution was prepared by reacting absolute ethanol (6 ppm H_2_O, 99%, Merck) with lithium metal (Albemarle Germany GmbH). Ethanol was dried over sodium metal and diethyl phthalate (99%, Merck), refluxed for 2 h, distilled and stored over 0.3 nm molecular sieve (Merck). For 0.5 M niobium ethoxide solution, Nb(OCH_2_CH_3_)_5_ (99.95%, Sigma-Aldrich) was dissolved in absolute ethanol. All of the synthesis steps were performed under an argon atmosphere. To prepare Li_2_CO_3_/LiNbO_3_-coated NCM622 cathode material, an amount of 5.94 g of NCM622 powder was added to 812 µL of niobium ethoxide solution. Next, the relevant amount of 1 M lithium ethoxide solution was added and the mixture was sonicated for 30 min at room temperature, followed by drying in a vacuum. The resultant powder was ground using mortar and pestle and then heated in a quartz tube furnace for 2 h at 300 °C in air (5 °C/min heating rate). Sample S6 was prepared following the same procedure, except that the final heating step was performed under oxygen flow (99.998%, Air Liquide). The uncoated (pristine) and coated NCM622 cathode materials were stored under an argon atmosphere.

### Preparation of electrode composites

Cathode composite was prepared by mixing an amount of 1 g of uncoated (pristine) or coated NCM622, β-Li_3_PS_4_ or Li_6_PS_5_Cl and Super C65 carbon black (Timcal) in a 7:2.9:0.1 weight ratio using 10 zirconia balls of diameter 10 mm for 30 min at 140 rpm in a planetary ball mill (Fritsch). Anode composite was prepared by following the same procedure, but using carbon-coated Li_4_Ti_5_O_12_ (NEI Corp.), β-Li_3_PS_4_ or Li_6_PS_5_Cl and Super C65 carbon black in a 3:6:1 weight ratio.

### Cell assembly and electrochemical measurements

First, solid electrolyte (60/100 mg for β-Li_3_PS_4_/Li_6_PS_5_Cl) was compressed at 125 MPa. Next, anode composite was pressed to the solid electrolyte pellet at 125 MPa and finally, cathode composite (~ 1.8 mAh/cm^2^ for 180 mAh/g_NCM622_) was pressed onto the other side at 375 MPa. A pressure of 55 MPa was maintained during the electrochemical testing of SSB cells. Charge/discharge measurements were performed at 25 °C and at a rate of C/10, with 1C = 180 mA/g_NCM622_, in the voltage range between 1.35 and 2.85 V vs Li_4_Ti_5_O_12_/Li_7_Ti_5_O_12_ (~ 2.9–4.4 V versus Li^+^/Li) using a MACCOR battery cycler.

### Attenuated total reflection-infrared (ATR-IR) spectroscopy

ATR-IR spectroscopy measurements were conducted in an argon glovebox using an ALPHA FT-IR spectrometer (Bruker), equipped with an Eco ATR sampling module. Acquired spectra were background corrected using the OPUS software (Bruker).

### X-ray diffraction (XRD)

Powder XRD measurements were conducted on the solid electrolytes sealed in a borosilicate glass capillary (Hilgenberg GmbH) using a STADI P powder diffractometer (STOE & Cie GmbH) with Cu-Kα1 radiation source. Rietveld refinements were performed using FullProf Suite. The Thompson-Cox-Hastings pseudo-Voigt function was used as a profile function. Scale factor, background coefficients (Chebyshev function with 24 parameters), lattice parameters and atomic coordinates (except for Li) were refined.

### Acid titration measurements coupled with mass spectrometry

The amount of carbonates (Li_2_CO_3_) on the uncoated (pristine) and coated NCM622 cathode materials was quantified by following a procedure already described in the literature^51^. The setup consisted of a septum-sealed vial containing a known amount of NCM622 powder, a mass spectrometer (HiCube Pro with PrismaPlus detector, Pfeiffer Vacuum) and a mass flow controller (F-201CV-020-RAD-33-Z). Carrier gas transporting the gaseous products from the vial to the mass spectrometer was flown through the vial via two PEEK tubings passed through the septum. The mass flow controller was used to set the flow rate of argon (6.0 purity, Air Liquide) at 2.5 mL/min. For the procedure, 1 M H_2_SO_4_ was injected into the vial through the septum with a needle. In each run, an amount of 10 mg of NCM622 cathode material was exposed to 0.35 mL of H_2_SO_4_ (degassed prior to use). Calibration gas was flown through the system after each run for CO_2_ quantification.

### Inductively coupled plasma-optical emission spectroscopy (ICP-OES)

The content of Li, Ni, Co, Mn and Nb in the uncoated (pristine) and coated NCM622 cathode materials was determined by ICP-OES using both a PerkinElmer Optima 4300 DV and a Thermo Scientific iCAP 7600.

### Transmission electron microscopy (TEM)

Scanning TEM (STEM) was conducted on the coated NCM622 cathode material using a Titan 80–300 microscope (FEI Company). The coating layer was protected by electron- and ion beam-induced Pt deposition prior to imaging (FEI Strata). TEM specimens were prepared by gallium focused ion beam (FIB) milling.

### Scanning electron microscopy (SEM)

SEM imaging was done at 10 kV using a LEO-1530 electron microscope (Carl Zeiss AG) with a field emission source.

## Supplementary Information

Supplementary Information.

## Data Availability

The datasets generated during and/or analysed during the current study are available from the corresponding author on reasonable request.
