# Supplementary material for: Effect of surface carbonates on the cyclability of LiNbO3-coated NCM622 in all-solid-state batteries with lithium thiophosphate electrolytes
Source: Sci Rep. 2021 Mar 8;11:5367. doi: 10.1038/s41598-021-84799-1 (PMC7940408; doi:10.1038/s41598-021-84799-1)
Supplement: Supplementary file 1 — Supplementary Information. [file 41598_2021_84799_MOESM1_ESM.docx]

Supporting Information

Effect of Surface Carbonates on the Cyclability of LiNbO_3_-Coated NCM622 in All-Solid-State Batteries with Lithium Thiophosphate Electrolytes

A-Young Kim,^1,^* Florian Strauss,^1,^* Timo Bartsch,^1^ Jun Hao Teo,^1^ Jürgen Janek,^1,2^ and Torsten Brezesinski^1,^*

^1^Battery and Electrochemistry Laboratory, Institute of Nanotechnology, Karlsruhe Institute of Technology (KIT), Hermann-von-Helmholtz-Platz 1, 76344 Eggenstein-Leopoldshafen, Germany.

^2^Institute of Physical Chemistry & Center for Materials Science, Justus-Liebig-University Giessen, Heinrich-Buff-Ring 17, 35392 Giessen, Germany.

*Email: [ayoung.kim@daimler.com](mailto:ayoung.kim@daimler.com), [florian.strauss@kit.edu](mailto:florian.strauss@kit.edu), [torsten.brezesinski@kit.edu](mailto:torsten.brezesinski@kit.edu)

**Table S1.** Atomic weight fractions from ICP-OES and acid titration measurements for the uncoated (pristine) and coated NCM622 cathode materials.

| Sample | Li / wt % | Ni / wt % | Co / wt % | Mn / wt % | Nb / wt % | LiNbO_3_ / wt %^†^ | Li_2_CO_3_ / wt %^‡^ | Total / wt % |
| --- | --- | --- | --- | --- | --- | --- | --- | --- |
| Pristine | 7.23 | 34.98 | 12.06 | 11.06 | <0.015 | – | 0.13 | 0.13 |
| S1 | 7.16 | 34.57 | 11.92 | 10.93 | 0.605 | 0.96 | 0.21 | 1.17 |
| S2 | 7.18 | 34.64 | 11.94 | 10.93 | 0.612 | 0.97 | 0.30 | 1.27 |
| S3 | 7.17 | 34.60 | 11.93 | 10.92 | 0.611 | 0.97 | 0.48 | 1.45 |
| S4 | 7.22 | 34.54 | 11.90 | 10.90 | 0.598 | 0.95 | 0.74 | 1.69 |
| S5 | 7.25 | 34.35 | 11.84 | 10.85 | 0.635 | 1.01 | 0.96 | 1.97 |
| S6 | 7.18 | 34.58 | 11.91 | 10.91 | 0.608 | 0.97 | 0.17 | 1.14 |

^†^Calculated based on the Nb content from ICP-OES (also for samples S1 and S2 despite the Li:Nb molar ratio being < 1.0). ^‡^Calculated using the amount of evolved ^12^CO_2_ from acid titration measurements.

**Table S2.** Refined lattice parameters for β-Li_3_PS_4_ (*Pnma* space group) from Rietveld analysis of powder XRD data (Fig. S3).

| Lattice parameter |  |
| --- | --- |
| *a* / Å | 12.982(4) |
| *b* / Å | 8.022(2) |
| *c* / Å | 6.117(2) |
| *V* / Å^3^ | 637.03(8) |

**Table S3.** Refined lattice parameter for Li_6_PS_5_Cl (*F*−43*m* space group) from Rietveld analysis of powder XRD data (Fig. S3).

| Lattice parameter |  |
| --- | --- |
| *a* / Å | 9.849(5) |
| *V* / Å^3^ | 955.38(1) |


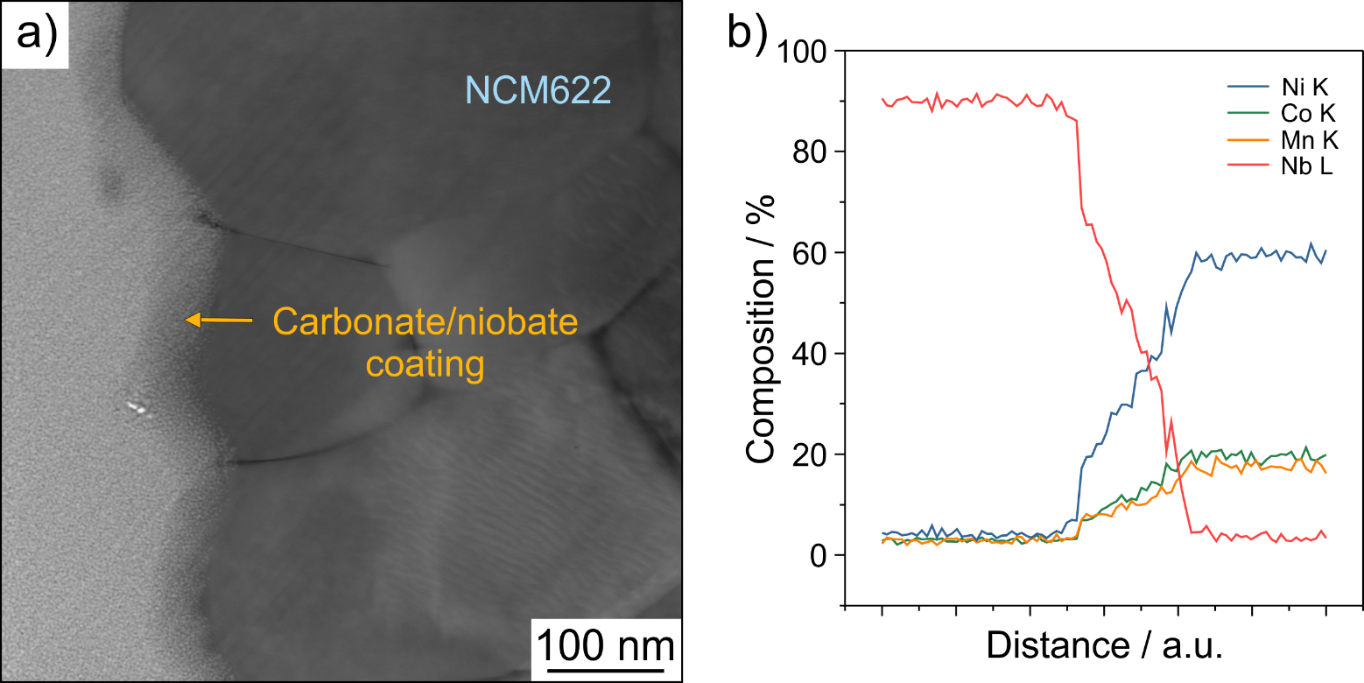


**Figure S1.** (**a**) High-angle annular dark-field STEM image of the coated NCM622 cathode material (sample S3) and (**b**) EDS line scan through the surface shell.


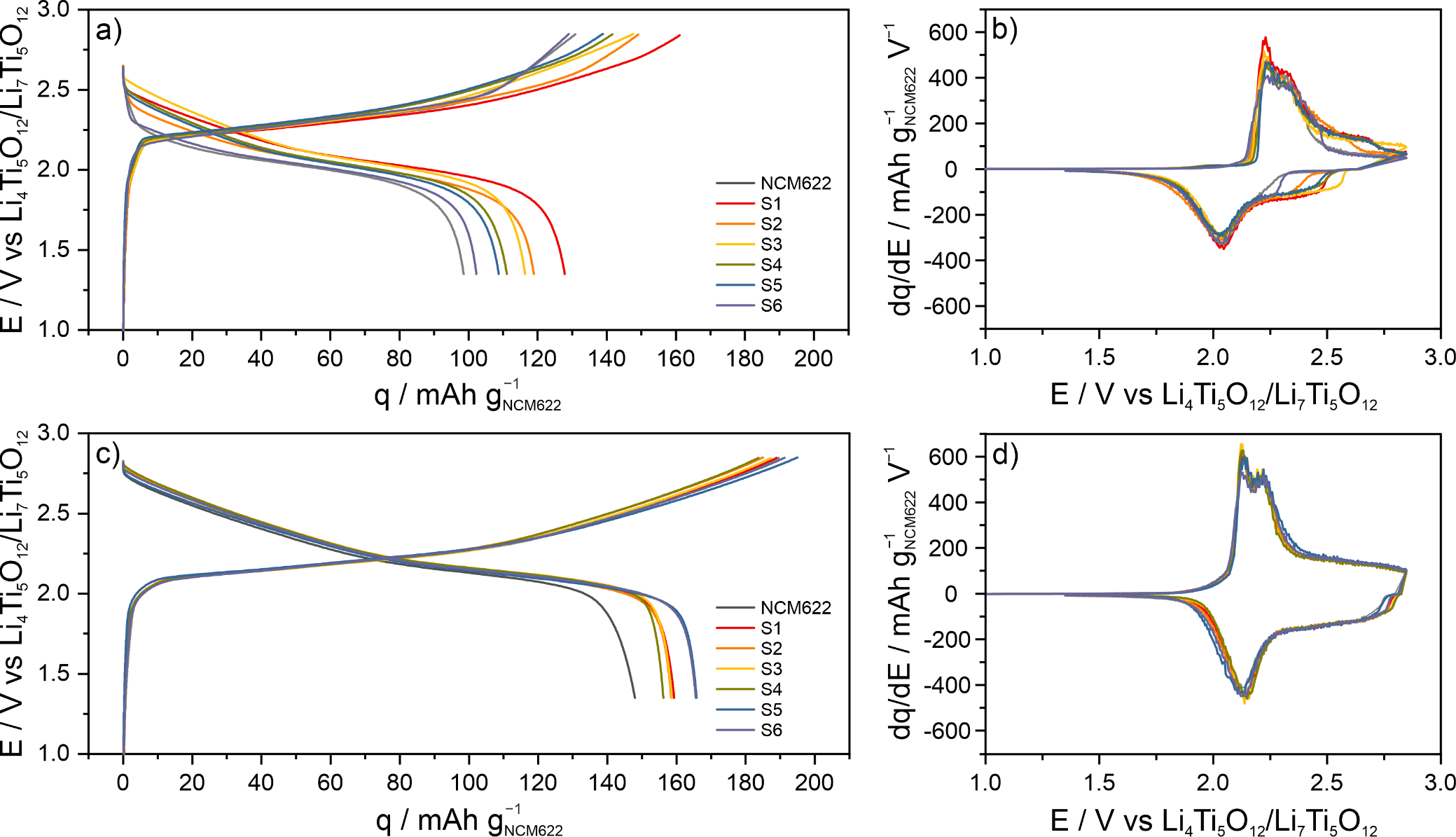


**Figure S2.** First-cycle charge/discharge curves at C/10 rate and 25 °C of pellet-stack SSB cells using the uncoated (pristine) or coated NCM622 cathode materials and (**a**) β-Li_3_PS_4_/Super C65 carbon black or (**c**) Li_6_PS_5_Cl/Super C65 carbon black. The corresponding differential capacity plots are shown in (**b**) and (**d**), respectively. The anode was made of LTO, β-Li_3_PS_4_ or Li_6_PS_5_Cl solid electrolyte and Super C65 carbon black additive.


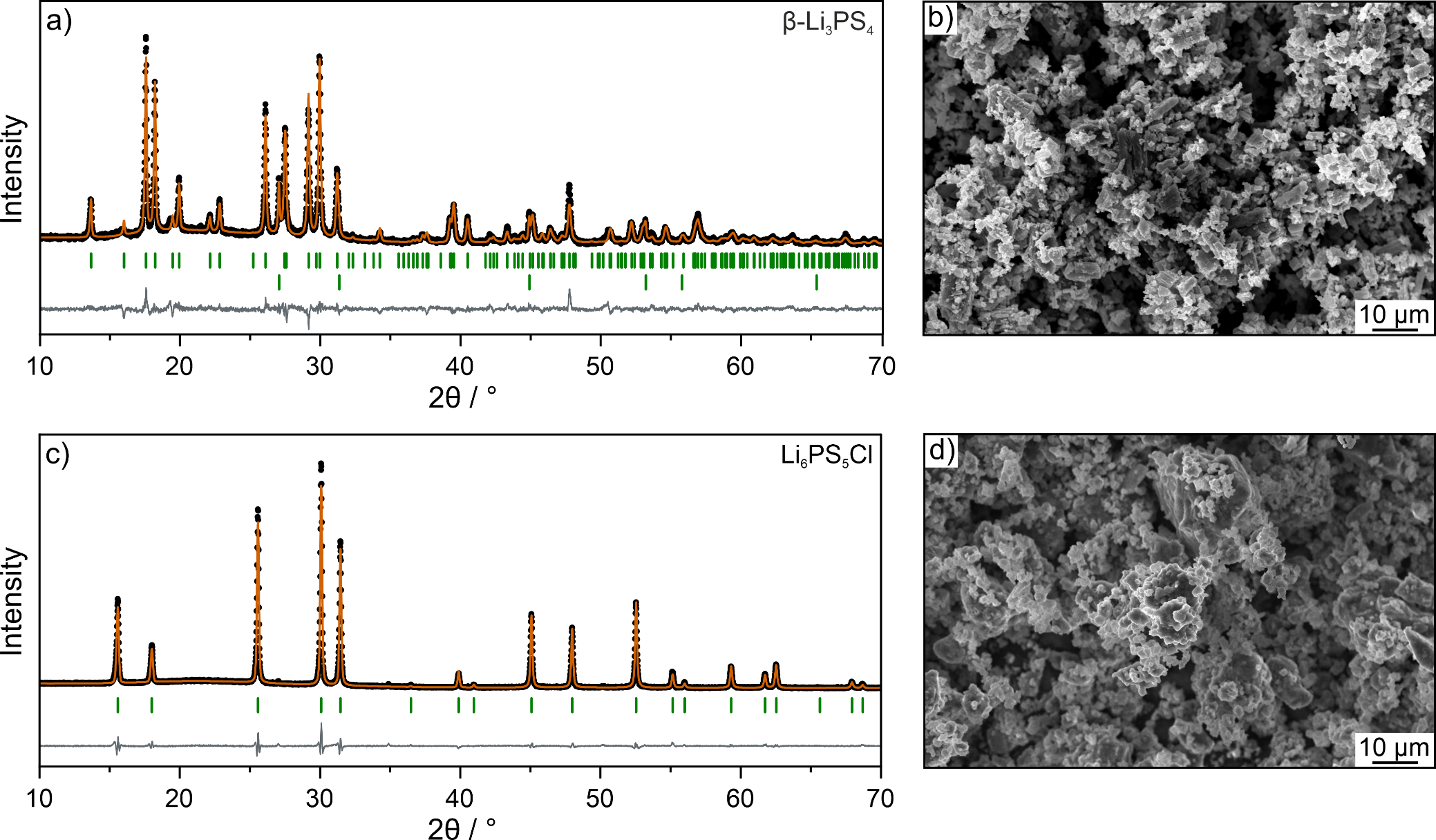


**Figure S3.** (**a**) XRD pattern of β-Li_3_PS_4_ and Rietveld plot for the refinement (*R*_wp_ = 8.49 %, χ^2^ = 1.97). Black circles and orange and gray lines represent the observed, calculated and difference profiles, respectively. Top and bottom (green) tick marks denote the Bragg reflections for β-Li_3_PS_4_ and Li_2_S (~8 wt % impurity), respectively. (**b**) SEM image of β-Li_3_PS_4_. (**c**) XRD pattern of Li_6_PS_5_Cl and Rietveld plot for the refinement (*R*_wp_ = 7.11 %, χ^2^ = 0.94). Black circles and orange and gray lines represent the observed, calculated and difference profiles, respectively. Green tick marks denote the Bragg reflections for argyrodite Li_6_PS_5_Cl. (**d**) SEM image of Li_6_PS_5_Cl. Refined lattice parameters are given in Tab. S2 and S3.
